# Private equity renewable energy investments in India

**DOI:** 10.1016/j.heliyon.2024.e41098

**Published:** 2024-12-14

**Authors:** Hemi H. Gandhi, Bram Hoex, Brett Jason Hallam

**Affiliations:** a School of Photovoltaic and Renewable Energy Engineering, University of New South Wales, Sydney, NSW, 2052, Australia; b Harvard John A. Paulson School of Engineering and Applied Sciences Cambridge, MA, 02138, USA; c Creative Synergies Group, Okemos, MI, 48864, USA

**Keywords:** India, Renewable energy, Private equity, Investment strategy, InvIT, Emerging markets

## Abstract

India is anticipated to grow its total energy consumption and CO_2_ emissions by more than any other country over the next two decades. India will have to attract around $400 billion in financing to realize its 500 GW target of renewable energy by 2030. Given complex renewable energy sector risks, rapidly scaling-up risk-friendly private equity financing will be critical to achieve India's target. This research seeks to answer questions regarding the motivation, perceptions, strategies, and investment behavior of private equity investors in the Indian renewable energy sector. The answers to these questions presented herein have been distilled from primary research interviews with 40 executive-level sector practitioners and literature analysis. This research finds that global-macro forces and sector-specific appeal are attracting varied investors to the sector. These investors primarily deploy capital in existing developer platforms, creating control developer platforms, and Infrastructure Investment Trusts. Critical investment criteria and value creation strategies of these investors are comprehensively discussed. Emerging investment opportunities to create new renewable energy sector value by partnering with companies across the power sector value chain are also presented. This research concludes that despite significant sector risks, investors remain confident they can achieve outsized risk-adjusted returns relative to most other global infrastructure assets. This optimism stems from the confidence of investors in their own ability to manage risk-return dynamics through judicious investment selection and management strategies, the sector's uniquely large demand growth and market size, and the Indian central government's perceived strong commitment to finding creative solutions to chronic sector issues. Lessons from the investment insights, themes and factor analyses discussed herein can be drawn upon in evaluating renewable energy investments and policymaking worldwide.

## 1 Introduction

### 1.1 India's 500 GW renewable energy target and financing challenges

India is growing through a stunning energy transition. Its population of 1.43 billion (bn)^1^ [1] is rapidly urbanizing and industrializing. Each year, India grows its urban population by a city the size of London [2]. Each year over the last decade it has connected 50 million new citizens to the electricity grid. India is already the 3rd largest emitter of annual CO_2_ emissions, but its average household electricity consumption is still just a tenth of households in the United Sates [2]. With its enormous population size and scope for future development, India is expected to grow its total energy consumption and CO_2_ emissions more than any other country over the next two decades [3]. Between 2021 and 2040, India is set to double its energy consumption and account for 25 % of total global energy demand growth [3].

Against this backdrop, India has taken global leadership in the fight against climate change.^2^ During the 2021 United Nations COP26 Conference on Climate Change, India's Central^3^ government (the Centre) announced [4] bold climate commitments to be achieved by 2030. These commitments include 1) installing 500 GW of renewable (RE) capacity, 2) meeting 50 % of the country's electricity generation from RE and 3) reducing total projected carbon emissions by 1 billion tons between 2022 and 2029 [5]. Rapidly scaling-up RE deployment offers India a pathway [4,6,7] to both decarbonize and meet its fast growing electricity consumption needs, which is expected to continue growing between 5.5 and 8.5 % annually over the next decade [8,9]. RE deployment offers India additional benefits compared to its historical reliance on imported coal: lowered electricity costs, smaller current account deficits, improved energy security and pollution mitigation [[3], [4], [5], [6], [7]].

Recent Indian RE literature has characterized the financial challenges of meeting India's 2030 climate commitments. At the end of 2022, India's photovoltaic (PV) and wind capacity stood at 100 GW. To meet both its climate commitments and growing electricity demand, India will need to add an estimated 320 GW of new PV and wind capacity between 2022 and 2029 [5]. This will require an estimated $220 bn in new-build PV and wind project financing between 2022 and 2029 [5]. The needed $220 bn investment is three times the $70 bn that flowed into new-build PV and wind capacity during 2014–2021 [5]. An additional $200 bn must be mobilized to concurrently modernize and equip India's grid and storage infrastructure to accommodate all the new generation capacity being added [5]. During the 2021–2022 fiscal year, the Indian RE sector (specifically for existing and new-built PV and wind) received $14 bn in financing spread across acquisitions (42 %), bonds (33 %), bank debt (13 %), equity (8 %) and joint ventures (4 %) [10]. To meet India's 500 GW target, sustained annual investment across the RE ecosystem (PV, wind, storage, grid) will need to more than double to $30–40 bn [10]. Clearly, RE financing—from all different sources—will need to be scaled up to meet this financing challenge.

Existing Indian RE sector literature has largely centered on characterizing the origin and nature of complex investment risks that limit capital flow [[11], [12], [13], [14], [15], [16], [17], [18], [19], [20], [21]]. Significant examples of such risks include the primary contractual power offtakers—State Distribution Companies (DisComs)—renegotiating payment terms of 20-year project Power Purchase Agreements (PPAs), delaying payments by as much as 540 days, or curtailing power to avoid payment [21]. Studies of sector funding activity have largely focused on bond and bank debt financing covering topics ranging from investment trends [3,22], Investor expectations [[22], [23], [24]], key risks [5,24], private and regulatory instruments to mitigate risks [15,[25], [26], [27], [28]], and strategies to broadening the investor pool [[28], [29], [30], [31]]. To date, however, comprehensive analysis of sector private equity (PE) financing has been lacking. This is a significant void for all of the following reasons since PE financing is a strategic and uniquely important source of financing for RE developers and their projects. First, PE is inherently risk and growth-friendly capital; it is well suited to the Indian RE sector's complex risks and fast growth trajectory. Second, equity financing is generally the prerequisite driver of other types of financing, and PE has become the dominant source of RE developers' project equity capital. 10.13039/100028163Equity initially motivates and supports developers to venture into new project. It is only after equity has been raised that developers negotiate project PPAs with offtakers and raise project debt financing. Furthermore, every $1 of project financing can mobilize $4 of debt financing, since projects are typically financed utilizing 20–30 % equity and leveraging with 70–80 % debt [23] Through this process, PE investment in a developer increases external confidence in the developer, helping it raise large amounts of low-cost debt and attract better talent. Third, PE financing is also faster and easier to raise than debt. Fast PE capital injections allow developers to invest and grow their businesses aggressively and opportunistically. Fourth, for a developer, PE investor active participation in strategic business decision-making and strong governance requirements can raise management, operational and reporting standards resulting in long-term value creation. And fifth, the potential pool of PE financing, particularly from global institutional investors focused on managing over $100 trillion in assets under management, is massively larger than what Indian domestic capital markets can provide [5,28].

Given the Indian RE sector's large financing needs and complex investment risks, rapidly scaling-up sector private equity (PE) financing will be critical for achieving India's climate commitments. There is certainly an opportunity for increased capital flow from new PE investors; as of 2022, eight of the world's ten largest pension and sovereign wealth funds had yet to make private equity investments in the sector [5].

### 1.2 Goal of this research

This goal of this research is to answer research questions (RQs) regarding the motivation, perceptions, strategies, and investment behavior of private equity investors in the Indian renewable energy sector. The full list of these RQs is listed in the following Methods section. Presented answers to the RQs have been distilled from primary research interviews with 40 executive-level sector practitioners and literature analysis. By empowering stakeholders with a comprehensive understanding of current private investor activity, the authors hope to 1) make the RE sector more readily understandable and accessible to potential new investors and 2) inform better policy making decisions to attract new investment.

This research proceeds as follows. First the RQs and methods employed in the research are presented. Next, the global-macro forces and specific benefits that are attracting global investors to the sector are described. The discussion is followed by characterizing the major categories of sector PE investors and their investment objectives. Analysis is then provided of the three most prevalent classes of sector PE investments: investments in existing developer platforms, creating control developer platforms, and Infrastructure Investment Trusts (InvITs). Investors' critical investment criteria and value creation strategies are comprehensively discussed. Following this, emerging investment opportunities to create new renewable energy sector value by partnering with companies across the power sector value chain are presented. This research concludes that despite significant sector risks, investors remain confident they can achieve outsized risk-adjusted returns relative to most other global infrastructure assets. This optimism stems from the sector's unique large demand growth and market size, combined with the Indian central government's perceived strong commitment to finding creative solutions to chronic sector issues. The investment insights, themes and factor analyses discussed herein are relevant to renewable energy investments and policymaking worldwide.

### 1.3 Scope of this research

This research analyzes sector PE investments in the context of Indian governmental regulation and PE market conditions until the end of 2021 when the authors' primary research interviews concluded. Figure data (obtained from private data sets belonging to industry practitioners) contains time series data ending during the 2019–2020 period; more recent data was not available. Sections 2, 3, 4, 5, 6, 7 of this research were also written with the intent of focusing on themes most relevant to RE investments in photovoltaic (PV) and wind generation, which are the largest non-Hydro sources of RE in India. Relatedly, the majority of sector experts interviewed were primarily involved in PV and wind project investments, with direct experience in these technologies. The research's themes and value creation strategies are likely broadly relevant to other emerging forms of RE technology (e.g., hydrogen, solar-wind hybrid plants, batteries, hydro, biomass), but do not shed insights specific to investments involving other emerging technologies. Readers interested in Indian RE topics beyond the scope of this research—debt investments [5,30,31], proposed regulatory reforms to mobilize new capital [3,15,19,21], investments in emerging technologies apart from PV and wind [[32], [33], [34], [35], [36], [37], [38], [39]]—are referred to cited recent publications on these topics. Readers seeking broader context for this research regarding how RE financing [40] and investment Environmental Social Governance (ESG) [41,42] factors influence progress towards the United Nations Sustainable Development Goals [43] In India [[44], [45], [46], [47]] and globally [48] under present forecasted climate change scenarios [49] are referred to cited recent publications on these topics.

While this research exclusively focuses on PE investments in the Indian context its broader global relevance should be noted. Lessons from the investment insights, themes and factor analyses discussed here can be drawn upon when evaluating RE investments and policymaking worldwide. While every country's RE market has undergone a unique growth trajectory and has its own challenges, the investor objectives, investment criteria and value creation strategies discussed herein are fundamental enough to be widely applicable to RE investments globally. Discussed investment strategies to specifically mitigate risks that are more idiosyncratic to India's context, however, will be most relevant to emerging markets with political-economic and power sector features similar to those of India. These salient features include a federalist form of government, a federalist and highly politicized power sector, high electricity demand growth, significant offtaker risk, large variance in offtake risk amongst government electricity companies and/or corporate buyers, and high project development risk. Applicable emerging markets with at least two of these features include Mexico [50], Nigeria [[51], [52], [53]], Pakistan [54], Iraq [55,56], Nepal [57], and Malaysia [58,59].

## 2 Materials and methods

This goal of this paper is to provide answers to the following research questions (RQs) pertaining to the motivation, strategies, perceptions, and the behavior of private equity investors in the Indian renewable energy sector.

1. Which global and domestic Indian PE investors are investing in the Indian RE sector?
2. Why do investors find the sector appealing?
3. How do investors compare the relative appeal of Indian RE investments versus other Indian Infrastructure asset classes?
4. How do investors compare the relative appeal of RE investments in India versus those in other countries?
5. What are the different types of sector investments that are being made?
6. Which sector investment risks do investors find the most significant?
7. Do foreign investors located outside India fully understand sector risks?
8. What strategies do investors use to mitigate investment risks?
9. What is the central government doing to attract RE investment?
10. How do investors perceive the central government's long-term commitment to RE investors?
11. How do investors perceive the central government's present and future ability to mitigate investment risks, particularly Power Purchase Agreement (PPA) contract breach risk?
12. What criteria do investors use when evaluating potential investment?
13. What strategies do investors use to create value in their investments?
14. What are emerging opportunities for investors to create new value in the sector?

To obtain answers to the RQs, the authors sought 1) empirical insights from a diverse set of sector experts via primary research interviews and 2) analytical insights from literature published in academic journals and industry research reports covering the Indian power sector and Indian RE finance. The authors conducted primary research interviews with 40 executive-level practitioners from PE investment firms, investment banks, project developers, Engineering Procurement Companies, major consultancies, and policy makers. Corporate interviewee positions ranged from Founder, CEO, former CEO, Managing Director, Vice President, Principal to Associate. Interviews were conducted by video or phone call—with interviewees located in India, the United States, Canada, United Kingdom, Switzerland, Singapore, and the U.A.E—between February 2020 to August 2021. Interview lengths ranged between 30 and 90 min and were conducted on the condition of anonymity. 13 interviewees were interviewed over 2–3 sessions; the rest were interviewed once. Interviewees were asked to express their opinion on the above RQs, their decision-making framework regarding specific investment transactions or policy legislations, and/or their past statements from prior published interviews. The authors requested an interview with a particular interviewee by “cold” contacting Interviewees by LinkedIn or email or were directly introduced to interviewees from previous contacts or other interviewees. The authors synthesized, categorized and distilled insights collected from primary research interviews and an analysis of published literature.

## 3 Renewable energy sector investment appeal

The combination of global macro trends and the sector-specific appeal that is attracting global PE investors to the Indian RE sector is discussed in this section.

### 3.1 Macro-trends driving investors to the Asian infrastructure opportunities

The following global investment macro-trends are driving investors to the Indian RE sector.

Unprecedented global liquidity and ultra-low yields: Fifteen years of unprecedented monetary and fiscal stimulus, following the Great Financial Crisis (GFC) and Covid-19 pandemic, led to historically high monetary liquidity and ultra-low real interest rates [61,62]. Concurrently, macroeconomic investment risks, including climate change, pandemics, terrorism, and political instability, have grown increasingly volatile and complex. Faced with a high-risk low real-yield world, investors have been forced to scour the globe for stable, higher-yield opportunities to deploy their excess capital in relatively higher risk environments [63].

**Fig. 1:**
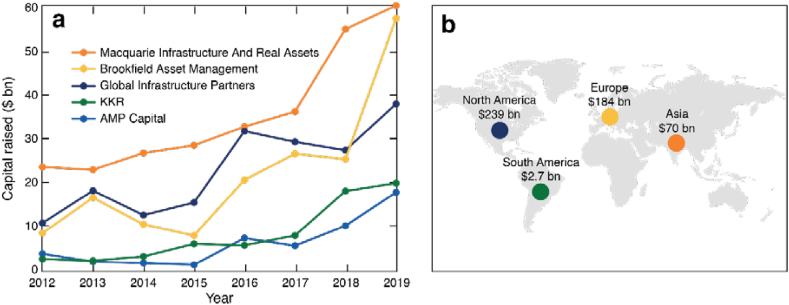
a) Annual capital raise by the world's five largest infrastructure PE firms b) Capital raise for deployment in select continents by the world's 50 largest infrastructure PE firms in 2019. Data from Ref. [60].

Growing global interest in infrastructure investments: Since the GFC, infrastructure investments have become increasingly valued as a stable, resilient, and predictable asset class that can weather economic downturns [63,64]. Institutional investors, like pension funds, rely on these investments to bring stable and consistent cash flows, to meet their cash obligations, regardless of the economic climate. Amidst this growing interest in infrastructure investing, the world's largest infrastructure PE firms have been able to raise larger amounts of capital every year as shown in Fig. 1a [60].

Growing interest in Asian infrastructure investments: PE infrastructure investment to date has been primarily concentrated in North America and Europe. Fig. 1b shows that in 2019, the world's 50 largest infrastructure firms raised 85 % of their total annual capital raise for deployment in North America and Europe [60]. Investors are now increasingly seeking to opportunistically diversify into underfunded Asian infrastructure, which is driven by tailwinds of population growth, urbanization, and rising incomes [65,66]. Several of the world's largest PE infrastructure investors have recently launched new Asia-focused strategies or distinct funds focused on India, Singapore, Taiwan, South Korea, Indonesia, andMalaysia [[66], [67], [68], [69]]. These countries have investment-grade sovereign credit ratings, respect for foreign investors' rights, perceived established sanctity of contracts, experienced infrastructure developers, and clear infrastructure need.

Perception of India as a top Asian-infrastructure-investment destination: Among the aforementioned Asian nations, investors perceive India to be uniquely attractive because of its large-scale market and significant economic growth potential. This scale advantage stems from its colossal 1.39 bn population, young demographics, rising incomes, massive urbanisation, growing electrification, large infrastructure deficit, and small domestic capital markets [64,66]. Investors also have confidence in the Centre's ongoing commitment to attract FDI for modernizing infrastructure.

ESG investor mandates: Stakeholder pressures are prompting investors to aggressively looking for opportunities compatible with Environmental Social and Governance (ESG) driven investing [[70], [71], [72], [73], [74]].

Interest in low-volume risk infrastructure assets: Macroeconomic uncertainty during and following the Covid-19 pandemic led several infrastructure investors to seek assets with cash flows unlinked to economic activity to avoid low-volume risks. RE assets meet these objectives because of their predictable and steady cash flows due to long term PPAs with fixed tariffs.

### 3.2 Appeal of Indian renewable energy investments

Within the Asian infrastructure investment opportunity set, Indian RE investments offer investors the following unique benefits.

**Fig. 2:**
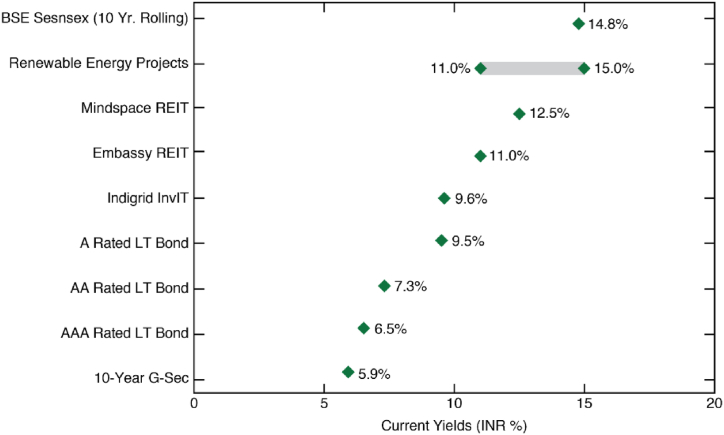
Returns across Indian asset classes. Returns as of October 2020. The Sensex 10-year rolling returns pertain to median returns over 2010–2020. An implied 5 % rental escalation has been added to the REITs' yield returns. Data from Ref. [75].

High, long-term yields: Operational brownfield assets typically provide USD 7–10 %^4^ (INR 11–15 %) annual returns through 25 year Power Purchase Agreements (PPAs) as shown in Fig. 2 [75,77,78]. Expected equity internal-rate-of-returns (EIRRs) in asset M&A transactions are typically between 9 and 11 % [79]. Expected IRR depends on offtaker strength. Lower risk assets with the strongest offtakers such as NTPC, SECI, and Gujarat yield returns at the lower end of this range, while higher risk assets with weaker offtakers, typically state DisComs, command higher returns [77]. Fig. 2 clearly shows the superior expected returns of RE assets relative to the country's leading publicly traded commercial Real Estate and Investment Trusts, the best performing InvIT and A-rated long-term bonds.

Sector maturity: Investors regard RE along with transmission lines and operating toll roads as the most mature infrastructure sectors [77]. Other sectors including waste management, water management, ports, airports, and rail do not currently offer the same regulatory predictability, established contracting framework, cash flow stability, and technology maturity to be considered “bankable” by most sophisticated investors [77]. The coal sector offers attractive scale but does not meet ESG mandates of institutional investors. Only RE, transmission, and toll roads have uniquely scaled up in India to sustainably absorb large swaths of investments [77].

Large market size: The Indian RE market ranks as the third-largest in the world and is expected to annually tender 8–10 GW [79] of RE projects. This allows investors and project developers to build large asset portfolios (1–10 GW+) and/or deploy large amounts of capital ($0.5 bn +) in single M&A transactions [77,78].

10.13039/100014345Strong Centre support Supporting the RE sector is a major strategic priority for the Centre. It is committed to promoting sector growth by aggressively addressing systemic issues and courting global investors. India made a significant commitment for RE in the early 2010s [17,80,81] and this was further accelerated under the current government (2016–present) [82]. The Centre's policy initiatives [83] combined with strong market dynamics has resulted in significant’ RE progress [84].

High liquidity: The RE sector has an active M&A market, with operational assets often traded a few years after commissioning, and 2–3 new entrants making direct investments every quarter. This high deal flow has several benefits. First, it increases investment liquidity and minimizes stranded asset risk^5^ [64]. Second, it allows investors managing new funds to do quick deals that demonstrate to limited partners that their fund strategy is promising and worthy of additional funds. Third, it provides fund managers with the confidence to invest in building experienced and knowledgeable sector investment teams, typically based in India or Singapore, to drive long-term objectives [64].

Low volume risk: RE has contracted steady cash flows from fixed-tariff PPAs. These offer investors stable, predictable cash flows with low volume risk. The Covid-19 pandemic has steered major investors away from previously popular toll-road opportunities and towards RE since toll-roads have volume-linked cash flows tied to travel activity. Although these volume-linked cash flows offer higher growth potential, they are vulnerable to significantly higher volume risk than RE PPAs with fixed-price tariffs. The uncertainty of the Covid-19 crisis made volume-linked assets hard to price and created large bid-ask spreads between buyers and sellers. As a result, RE investments have become more attractive, given their substantially lower uncertainty and ease of risk quantification.

ESG-fit: The Centre's bold global climate commitment and 500 GW RE target, make Indian RE investments highly congruent with ESG-investment-mandates.

### 3.3 Investor perceptions of sector risks

Indian RE investments are exposed to significant complexity and risks [21,26,85]. For example, long construction delays are very common and typical PPAs do not feature any protections for inflation. The primary offtakers are perennially financially distressed, State Distribution Companies (DisComs), that have an average payment delinquency of 11 months [86]. DisComs have reneged on billions of dollars of their commitments by cancelling 25-year PPAs [16]. Contract risks [16,87] are further exaggerated due to the very slow pace of the Indian legal system [88], which typically takes four years for contract enforcement, compared to one year in the U.S. The following nine categories of sector investment risks are the most critical [21]: Project Development Risks, Offtaker Risks, Stranded Asset Risks, Volume Risks, Curtailment Risks. Regulatory Risks, Inflation Risks, Exchange Rate Risks, Tail Risks.

The most significant risk, is offtaker Risk [18,21,23,26,89], where offtakers breach their contractual obligations by delaying/cancelling PPA signing, delaying payment and renegotiating/cancelling PPA. DisComs pursue these tactics due to their financial distress exacerbated due to falling RE tariffs [21]. Furthermore, routine breach of contracts is incentivized as a result of poor contract enforceability and a judicial system that is effectively dysfunctional [21]. Offtaker Payment Delay Risk is the most dominant risk in RE projects. It is the risk that payment for the contracted electricity will not be paid in full or on time. Cash starved DisComs routinely delay payments to preserve cash flows and reduce the high cost of working capital loans [21]. Although contractual payment time is 30–60 days, RE generators typically face average payment delays of 11 months [86]. These delays stress the liquidity of generators and compromise their ability to make debt payments on time. These adverse conditions coerce generators into obtaining high cost working capital loans which have a negative effect on their EIRRs and competitive positioning in the marketplace.

To mitigate offtaker risk and increase the confidence of investors, the Centre has empowered two Centre-owned for-profit companies to serve as intermediaries for power procurement on behalf of credit-risky DisComs. These companies—NTPC and the Solar Energy Corporation of India (SECI)—are typically referred to as “central offtakers.” In their RE power aggregation and trading business model, these companies orchestrate competitive reverse–bidding auctions [82,90] with developers and usually procure electricity through 25-year PPAs. The contracted power is sold to DisComs through Power Sale Agreements (PSAs) at reasonable trading margins of Rs 0.05–0.07/kWh which provide a cushion against DisCom payment delays/defaults [91]. NTPC/SECI generally sign PPAs with developers only after DisComs complete PSAs for the contracted power [91] in order to adequately insulate themselves from execution and market risk. Developers strongly prefer to contract with central offtakers, who have now significantly marched ahead of state DisComs in auction capacity volumes [22].

Central offtakers are perceived as very bankable for three primary reasons [21]. Investors primarily view NTPC/SECI PPAs as quasi-sovereign entities due to their sovereign heritage and their strategic positioning in the sector. Additionally, both NPTC/SECI have a proven track record of timely payments and an impeccable reputation with no instances of contract breach. Reported payment days from NTPC and SECI to solar IPPs—30 and 75 days, respectively—are substantially lower than payment days from most state DisComs, and starkly lower than the notorious Tamil Nadu and Andra Pradesh DisComs (540 days) [92]. Third, their PPAs feature strong payment security mechanisms [18,21,91] to provide protections against DisCom payment default.

In spite of the aforementioned risks, the Indian RE sector is still very attractive and able to draw billions of dollars in investment from existing/new investors regularly. Why are investors and businesses deploying substantial capital in the Indian RE sector in spite of all the risks?

Our primary research insights [21] suggest that despite significant risks, investors are deploying significant capital due to their strong confidence in the sector's fundamental underpinnings, as summarized below.

• India will have long–run electricity demand growth.
• India will have robust demand driven by policy and market forces in the long run for RE (8–10 GW/year) [79,93].
• The Centre is committed to resolving RE sector issues.
• Central offtakers are bankable intermediate procurers, despite the significant problems with the DisCom counterparties.
• The Indian judiciary is committed to the rule of law and will uphold contracts in the long run.

The Indian RE sector offers distinct value with project EIRRs of 7–10 % USD, significant market size, high level of deal liquidity, and an attractive ESG fit [77].

## 4 Categorical private equity investors active in the Indian renewable energy sector

### 4.1 Private equity investor categories and objectives

Seven categories of PE investors active in the Indian RE sector and their broad investment objectives are presented in this section. These categorical investors, listed from highest to lowest amount of sector capital deployed, consist of Institutional Investors, Private Infrastructure Funds, Government Development Funds, Global Oil and Gas Corporations, Generalist PE Funds, Private Impact Funds, and High-Net-Worth-Individuals (HNWIs).

Institutional Investors: Large direct Institutional Investors [94] view investing in Indian RE as an opportunity to deploy sizable capital, typically $0.5–2 bn, in long-term stable assets that simultaneously meet ESG obligations. Stable, long-term asset cash flows are deployed to meet their long-term cash obligations such as paying pensioners, for example. Developer equity interests also give them growth potential. To date, Canadian, Arabian Gulf, and Singaporean direct Institutional Investors have been most active in the sector. These investors invest “directly,” by making investments themselves versus indirectly investing in private PE firms that make investments on their behalf.

Private Infrastructure Funds: Large Private Infrastructure Funds are attracted to the Indian RE sector because it can absorb large-sized investments with high-deal flow. Sector size, deal flow, and liquidity allow them to quickly build a track record of initial successful exits and launch new funds. Such funds invest across Asia, only in India, or specifically in energy infrastructure worldwide.

Government Development Funds: Government Development Funds seek to target sector-transformative investments in emerging markets with high carbon-emissions and/or thin capital markets. They look for opportunities where they can mobilize additional private sector capital and deliver innovative capital structuring solutions for creating new market value, significantly lower cost of capital, bring in new investors, and unlock capital to make fresh investments in new projects. They generally make minority equity investments. These are typically administered within foreign or domestic finance institutions, primarily headquartered in the U.K., U.S. and India.

Global Oil and Gas Corporations: Large Oil And Gas Corporations want to enter the sector for resource diversification, growth opportunities in India's large market, obtain a source of stable cash to soften the volatility in their oil and gas business, and address shareholder ESG pressures [95]. Oil And Gas Corporations are culturally attuned to thinking of their investments with very long-term (50–100 year) views of energy markets. The entrance of these global and domestic Oil and Gas corporations observed here is a new sector feature not previously observed in older interview-based studies [23] of sector participants.

Generalist Private Equity Funds: Generalist PE funds look for sector-agnostic growth opportunities across Asia or South-Asia.

Private Impact Funds: Private Impact Funds have similar objectives to Governmental Development Funds, but expect higher equity returns and make smaller-sized investments.

High-Net-Worth-Individuals: Domestic Indian HNWIs invest in RE assets, typically a pool of assets versus equity in developers, for stable, fixed income cash flows to complement their growth investments in their family businesses or public equities. HNWIs invest in the sector via family offices or private wealth management divisions of investment banks such as Kotak-Mahindra, Avendus Capital, and Barclays for example. Indian HNWIs’ real asset exposure has been historically limited to real estate, land investments, and physical gold. In sharp contrast to real estate/land which have suffered large valuation declines after Indian demonetization [96], RE assets have the significant advantage of price stability. RE assets are also very attractive for domestic HNWIs as compared to gold since they offer the benefit of substantial cash yields. Recent years have seen growing activity of impact-oriented, international HNWIs investing in RE and sustainability-focused businesses and projects worldwide [97]. While this contingent has not significantly invested in India RE sector to date; their capital pool represents an important potential capital pool for the sector moving forward.

### 4.2 Long-term owners versus medium Term Investors

The aforementioned investor categories are subsumed within either one of two broad categories: Long-Term Owners and Medium-Term Investors. Long-Term Owners intend to hold equity in a developer or asset portfolio for 10–25 years. In contrast, Medium-Term Investors intend to invest in growing developers or asset portfolios for 5–10 years and then exit through selling their equity to long-term owners. Direct Institutional Investors, global Oil and Gas Corporations, and HNWIs are typical long-term owners. These investors generally look for annuity-like cash flows from stable, high-quality operational assets that also have equity growth potential. Medium-Term Investors are usually Private Infrastructure Funds, Development Funds, Generalist PE Funds, and Private Impact Funds.

### 4.3 Investment examples

Illustrative examples of such categorical investors and their recent investments are shown in Table 1.

**Table 1:** Illustrative examples of such categorical investors and their recent investments [[98], [99], [100], [101], [102], [103], [104], [105], [106]] are shown in Table I.

Investor Type	Investor	Headquarters Domicile	Investment	Investment Type	Investment Amount∗ (USD)
Sovereign Wealth Fund	Abu Dhabi Investment Authority (ADIA)	UAE	ReNew Power (NYSE-listed)	Utility-Scale Developer	$300+ million (mn)
Sovereign Wealth Fund	Abu Dhabi Investment Authority (ADIA)	UAE	Greenko Energy Holdings	Utility-Scale Developer	$2.3 bn (Collectively with GIC)
Sovereign Wealth Fund	Mubadala Investment Company owned Masdar Clean Energy	UAE	Hero Future Energy	Utility-Scale Developer	$150 mn
Sovereign Wealth Fund	Qatar Investment Authority (QIA)	Qatar	Adani Electricity Mumbai	Private DisCom	$450 mn
Pension Fund	CDPQ	Canada	Azure Power (NYSE-listed)	Utility-Scale Developer (Majority Stake)	$320 mn
Sovereign Wealth Fund	GIC	Singapore	Greenko Energy Holdings	Utility-Scale Developer (Majority Stake)	$2.3 bn (Collectively with ADIA)
Sovereign Wealth Fund	GIC	Singapore	IndiGrid InvIT	Utility-Scale Control Platform and InvIT	$140+ mn
Sovereign Wealth Fund	Temasek	Singapore	I Power	Utility-Scale Control Platform	$500mn (with EQT infrastructure)
Infrastructure Fund	KKR	U.S.A.	IndiGrid InvIT	Transmission/PV InvIT	$360+ mn
Infrastructure Fund	KKR	U.S.A.	Virescent Infrastructure And Virescent Renewable Energy Trust InvIT	Utility-Scale Control Platform and InvIT	N/A
Infrastructure Fund	Global Infrastructure Partners (GIP)	U.S.A.	Vector Green Energy	Utility-Scale Control Platform	N/A Looking to sell platform for $700 mn
Infrastructure Fund	Eversource Capital Green Equity Growth Fund (GEEF)	India	Ayana Renewable Power	Utility-Scale Control Platform	N/A
Infrastructure Fund	Eversource Capital (GEEF)	India	Radiance Renewables	Utility-Scale Control Platform	N/A
Infrastructure Fund	Actis Capital	U.K.	Ostro Energy	Utility-Scale Control Platform (Wind)	$280+ mn
Infrastructure Fund	Actis Capital	U.K.	Sprng Energy	Utility-Scale Control Platform (PV)	$450+ mn
Infrastructure Fund	EQT Infrastructure	SwedeIO2 Power	Utility-Scale Control Platform		$500 mn (with Temasek)
Government development fund	UK Climate Investments (UKCI)	U.K.	CleanMax Solar	C&I Developer	$40–60 mn
Government development fund	CDC	U.K.	Ayana Renewable Power	Utility-Scale Control Platform	$70+ mn
Government development fund	National India Infrastructure Fund (NIIF)	India	Ayana Renewable Power	Utility-Scale Control Platform	$284+ mm
Oil and Gas	Shell	Netherlands	Cleantech Solar	Utility-Scale Developer (Pan-Asia/India) (49 % Stake)	$100 mn
Oil and Gas	Shell	Netherlands	Orb Energy	PV Rooftop Developer (20 % Stake)	N/A
Oil and Gas	BP (Light Source BP)	U.K.	Ayana Renewable Power	Utility-Scale Control Platform	N/A
Oil and Gas	Petronas	Malaysia	Amplus Energy Solutions	C&I Developer (100 % Acquisition)	$375 mn
Oil and Gas	Total	France	Adani Green Energy (BSE–listed)	Utility-Scale Developer (Vertically integrated)	$2.5 bn
Generalist PE Fund	Goldman Sachs	U.S.	ReNew Power	Utility-Scale Developer	$470+ mn
Generalist PE Fund	Warburg Pincus	U.S.	CleanMax Solar	C&I Developer	$60 mn
Impact Fund	TPG Rise Fund	U.S.	Fourth Partner Energy	PV Rooftop Developer	$70+ mn
High Net Worth Individuals	Das & Co Family Office	U.S./India	SuryaDesh Energy	Control-Platform	N/A

## 5 The three renewable energy sector investment classes

PE investors primarily deploy their capital in three preeminent classes of sector PE investments: investing in existing developer platforms, creating new control developer platforms (henceforth “control platforms”), and investing in or creating Infrastructure Investment Trusts (InvITs). Each investment class will now be comprehensively discussed.

### 5.1 Role of developers

The first two aforementioned investment classes involve RE developers.

Developers plan and build new RE plants, and often own and operate the plants they build or acquire. In this role, they function as Independent Power Producers (IPPs) since they privately produce and sell electricity to central offtakers, DisComs or private businesses or households. In this work, the term developer refers to IPPs. Developers bid for project PPAs from the government, commercial, or residential customers and build/operate RE assets to supply the contracted electricity. They take greenfield project development risk and obtain project permits, procure land or roof space, set up transmission grid connectivity, and are responsible for plant construction. Large developers typically have in-house Engineering Procurement and Contracting (EPC) teams that source plant hardware, and design and build plants. Developers are generally focused on either utility-scale projects or distributed-scale projects, although some operate in both segments. Distributed-scale developers primarily focus on serving corporations with A to BBB credit-ratings [107] through rooftop PV projects or land-based group-captive open access projects.

### 5.2 Investing in existing developer platforms

When PE investors, often “long-term asset owners, invest in an existing developer platform they are backing the existing management to grow the business. The management team uses PE investors’ equity capital to improve and grow their asset portfolio and business operations. When direct institutional investors invest in an existing developer platform, they invest with a long 15–20 year time horizon. It is often important for institutional investors to invest enough capital such that they have equity rights to board seats that will give them reasonable overview and/or control over management decisions. For example, Canadian pension fund CDPQ made their first investment in leading solar developer Azure Power in 2016, made several follow-on investments over a four-year period, and now has a majority controlling interest in the developer [99].

### 5.3 Creating new control developer platforms

When a PE fund creates a new control developer platform, they are making a much more hands-on entrepreneurial investment. These investors build the platform from scratch and usually have a majority control interest in the platform from the date of incorporation. They incorporate the business, hire the management team, actively engage in operations and help the management team acquire existing operational assets to build the portfolio. Investors make all of the platform's investment and management decisions with the goal of creating a high-quality asset portfolio that will be attractive to a future buyer, most likely an existing large developer or a long-term PE owner.

### 5.4 Infrastructure Investment Trusts

InvITs are the third type of prominent Indian RE PE investment. An InvIT is a special purpose investment vehicle for Indian infrastructure investing [108,109]. The Securities and Exchange Bureau of India (SEBI) created the InvIT structure to attract long-term FDI to the infrastructure sector [109].

An InvIT acquires, owns, and operates a pool of long-term, stable, cash flow generating assets. InvITs collect capital from investors, InvIT unit holders, to invest in infrastructure assets. InvITs operate similarly to REITs [108] and YieldCos [110], with specific regulatory requirements optimized for the Indian infrastructure sector.

SEBI created the InvIT structure to make infrastructure investing in India similar to that in OECD countries: investing in an operating cash flow/yield generating asset class versus investing in riskier, development assets. The InvIT structure is well suited to the investment objectives of long-term institutional investors that seek long-term, diversified and predictable cash flows to match their long-term cash obligations [109]. The structure also makes it easier for HNWIs and retail investors to invest in infrastructure through fractional ownership in listed InvITs. The typical InvIT investor IRR return expectation is between USD 6–10 %, depending on the risk perception of the infrastructure asset category.

SEBI provided initial regulatory guidelines for InvITs in 2014 [109]. As of November 2021, 15 SEBI-registered InvITs operate across digital fibre, roads, transmission and RE [111]. Among these, the following three are relevant to this work. India Grid Trust (IndiGrid, publicly listed 2017) focuses on transmission and utility-scale solar assets [112,113]. Powergrid InvIT (PGInvIT), publicly listed in 2021, focuses on transmission [114] and Virescent Renewable Energy Trust (VRET), privately listed in 2021) [115], focuses on utility-scale solar assets. Analysts estimate that InvITs can potentially raise up to $100 bn in infrastructure financing (across sectors) over the next five years [116].

## 6 Critical renewable energy investment criteria

One of the most important objectives in our primary research was to thoroughly understand the critical investment criteria for the RE sector by interviewing leading sector investors, investment bankers, developers, consultants, and policymakers. Our distilled insights clearly suggest that investors and businesses are primarily driven by the following seven critical investment criteria to evaluate the discounted future cash flows of potential RE sector investments.

### 6.1 Management team strength

Strong management teams will skillfully generate business growth and mitigate sector risks. Hallmarks of strong management teams are deep experience in the Indian power sector and a proven track record of successful project execution, well-articulated business vision/plan and well-established relationships with policymakers and regulators. Managers with close ties to politicians and regulators can positively influence the development of business-friendly regulations. Institutional investors, who plan on investing and reinvesting in developer platforms over a 10–20-year period, want managers who have a long-term vision for the business and are nimble enough to compete in the short and medium term. Institutional investors, who have ESG investing imperatives, also seek management teams with a demonstrated track record of strong corporate governance, frequent reporting, and decision-making transparency [117]. Several institutional investors have publicly detailed criteria of what they look for in business partners [73,118]. PE investors who create control-developer platforms from scratch, look to hire management executives with a record of successful exits.

### 6.2 Business plan strength

Investors critically evaluate a developer's business plan and market assumptions. This process assesses the developer's positioning, expertise, understanding and insightful planned adoption of emerging technologies, appetite for equity, and ability to compete with the broader market given assumptions about market RE tariff trajectories. To sustainably create value, strong utility-scale platforms must have the ability and plans to raise large amounts of low-cost debt, execute new projects on time, effectively liaise with policy makers, offtakers, and regulators, have high-quality relationships with suppliers, have in-house EPC and O&M capabilities, and a lean corporate structure/overheads. Likewise, strong C&I platforms must have the ability and plans to establish a distributed localized presence throughout India and neighboring nations, grow a reputable franchise/brand recognition, liaise with project stakeholders demonstrate customer retention and repeat order capabilities, high-quality execution, opportunistically raise equity capital for large Open Access projects and geographic expansion, in-house EPC/O&M capabilities, and a lean corporate structure/overheads.

### 6.3 Asset portfolio quality

Investors typically evaluate portfolio quality via four metrics: plant quality, approvals and permits, PPA quality, and portfolio diversification.

Plant quality: High-quality RE plants will produce optimized amounts of electricity throughout their life. Well-constructed power plants, with high-quality, well-maintained hardware, situated in locations of rich solar or wind resources with clear grid access, will generate larger amounts of delivered electricity. The strongest PV projects operate with plant availability factors greater than p-90 generation estimates, 97 % plant availability, and 97 % grid availability [119]. Higher cash flows from increased electricity generation enhance asset owner equity returns. Plants should have access to reliable sources of water, as water cleaning is critical for both wind turbine and PV performance [21,120]. Investors should also deploy robust technical advisors who can make multiple visits to the plant site for technical assessment to ensure high-quality generation for the next 15–25 years. Compared to a 2016 study [23] interviewing RE developers and financiers, our findings suggest that concerns about RE plant technology and construction risks have substantially reduced over the last eight years.

Approvals and permits: Greenfield RE projects require a large number of approvals and permits. Investors are careful to ensure that a developer, its contractors and suppliers have rigorously followed all approvals/permits before proceeding with constructions. Critical among these are land-use and transmission/distribution rights. In general securing such rights can be particularly difficult for wind projects [121]. Greenfield projects in Central and State solar or wind parks, however [122], benefit from pre-secured land-use and transmission/distribution rights. Furthermore, investors seek to ensure that management teams operate with high integrity and follow all Indian laws so as not to put investors in legal jeopardy within their home countries. Otherwise, American investors, for example, could fall in violation of the Foreign Corrupt Practices Act [123].

PPA quality: PPA quality determines the contracted cash flows power producers expect to receive from offtakers. Investors evaluate PPA quality based on three metrics: off-taker strength, tariff prices, and tenor. PPAs with stronger, more credible offtakers are more likely to result in predictable cash flows, than PPAs with riskier offtakers. The dominant strategy for minimizing payment risk is to sign/buy PPAs with offtakers, who are financially strong with a clean history of timely payments. The strongest offtakers in the utility scale market are SECI, NTPC and Gujarat DisComs, whereas A+ rated Indian and global corporations are the strongest offtakers in the C&I market. Due to the lower counterparty risk PPAs with stronger offtakers, yield lower electricity tariffs. Central offtaker auctions yield lower tariffs with 200 bp lower EIRRs than DisCom auctions [22]. Typically in M&A transactions, investors are willing to pay a premium for assets backed by SECI/NTPC PPAs due to the high level of certainty for timely payments [79]. This is one of the primary finding of this work. It should be noted that this large-scale successful demonstration of India's central offtaker mechanism validates prior theoretical studies from the early 2010s [17,124] that advocated the policy creation of central government Payment Security Mechanisms as a means of increasing sector private investment.

Most investors avoid acquiring older RE assets despite of their high tariffs due to the high probability of stranded asset risk. Furthermore, investors and IPPs mitigate this risk by exiting older assets to unlock their capital and transferring the stranded asset risk to new buyers. Multiple investors interviewed stated that in 2021 they were not willing to consider buying assets with tariffs above 4.0 INR/kWh to stay away from stranded asset risk – whereas prior to reverse auctions (around year 2017), they would have readily acquired RE assets with PPA tariffs between 7.0 and 10 INR/kWh.

Portfolio diversification: Diverse portfolios with assets contracted with diverse offtakers will have reduced idiosyncratic offtaker risk compared to portfolios with concentrated offtakers. Diversification will also enable investors to opportunistically allocate a portion of their portfolios to higher return assets with higher tariff PPAs with weak offtakers while minimizing aggregate portfolio risk.

### 6.4 Corporate financial structure flexibility

Businesses with well-designed corporate financial structures allow management teams to extract the maximum amount of cash out of an asset holding structure and use this cash at their discretion. It is important for businesses to have well-designed financial structures, because Indian corporate finance law makes it difficult to undo and re-organize existing financial structures without paying significant tax penalties or violating the law. For example, it is not possible to change a convertible debenture to a non-convertible debenture, to take more cash out. Well-designed corporate finance structures will allow the management to extract as much cash as possible out of the SPV and Hold-Co and deploy it per their discretion. Well-designed structures will allow management to move Hold-Co expenses down to SPVs to free-up Hold-Co cash, move SPV cash up to the Hold-Co, and use cash appropriately to grow the business.

### 6.5 Balance sheet strength

Businesses with strong balance sheets will be able to attract lower-cost capital and service a healthy amount of leverage. Investors must ensure that the business has adequate cash flows to service debt and be resilient in the event of potential liquidity issues due to payment delays. Furthermore, the business must not be overly leveraged and have adequate margins of safety to respond robustly in adverse economic climates. Since operating profitability is a critical component of a business's cash flows, it is important to ascertain that operational expenses, which account for 88–92 % of revenue, are in line with industry metrics. The strongest PV developers typically operate with total debt/OPBITDA ratios less than 1.5, cumulative DSCR/debt tenure greater than 1.5, and a minimum DSCR/debt tenure ratio greater than 1.35, and IRRs in excess of 12 % [119].

### 6.6 Complement to existing investment portfolio

Investors seeking to build a diversified asset portfolio, will look to acquire assets that complement their existing holdings. Complementary variables include: offtaker type, generation source and geography. Portfolios diversified across these variables will not only have reduced idiosyncratic risk but also be well positioned to effectively compete in new procurement models [81] like round-the-clock PPAs and solar-wind hybrid PPAs.

### 6.7 Potential to catalyse sector transformation

Government development and private impact funds typically prefer to deploy capital in undercapitalized sub-sector areas to catalyse their growth. In India, they have to date focused on green-field project development and distributed renewable development in the C&I and residential rooftop subsectors.

## 7 Renewable energy investment value creation strategies

This research will now discuss investment value creation strategies that investors employ to maximize sector returns. These strategies are most relevant to hands-on investors who seek to actively drive investment returns in control-platform investments. In such cases, managers aggregate a collection of operational RE energy assets to create a new asset portfolio. By employing different value creation strategies, the manager's objective is to maximize the value of the resulting aggregated asset relative to the sum of its parts.

Each of the following value creation strategies will be discussed, with particular detail give to financial engineering strategies.

### 7.1 Providing growth capital

The most common and basic way investors add value to a RE business is by providing the capital to grow the business in exchange for equity. Developers can use this equity capital to pay off existing debt, develop new assets, acquire new assets, invest in new offerings and capabilities, and expand into new markets. Developers can quickly raise large amounts of capital via equity financing rather than painstakingly raising debt from domestic Indian banks, which are currently severely financially stressed and wary of infrastructure lending.

### 7.2 Buying distressed assets below intrinsic value

Amidst the ongoing sector consolidation described above, investors encounter opportunities to buy distressed assets from developers looking to raise cash (possibly very quickly). In such a case, investors may be able to acquire the asset at a fraction of its intrinsic value. Developers positioned strongly enough to be able to choose which assets to sell often choose first to sell their oldest high-tariff PPAs assets to mitigate their stranded equity risk. Developers, who are extremely financially stressed, may be forced to sell their highest-quality assets first.

### 7.3 Attracting a high-quality management team

By creating an asset portfolio with larger total EBITDA than individual component assets, investors can incentivize and attract a better high-quality management team with a proven track record to more productively manage assets.

Improve business strategy: Investors can work with management teams to improve their business plans and refine their market assumptions. In this process, investors can utilize insights from their market observations, deal experience, networks, and accumulated RE firm learnings across geographies and industries.

Reduce costs via economies of scale: Investors can reduce management costs, overhead, redundant costs and get pricing discounts across the asset portfolio through economies of scale. In a recent example, PE investors running a control-developer-platform, acquired several PV assets, cut all in-house O&M, and outsourced O&M activities to a third-party contractor. Pricing discounts can also be obtained on land, hardware and outsourced EPC work across all portfolio development projects.

### 7.4 Optimizing operations and maintenance

Optimizing asset O&M can improve asset electricity generation and longevity. This will ultimately lead to increased cash flows via selling larger volumes of generation and creating the ability to service higher debt. Common examples of optimizing O&M include deploying smart technology to monitor operations, predictive maintenance and regular cleaning of PV panels.

### 7.5 Portfolio aggregation effect

A large, diversified asset portfolio, created through skillful acquisitions and some in-house project development, can command a larger EBITDA valuation multiple than individual components. Large, high quality, diversified portfolios are rare. They can be attractive to large institutional investors/asset-owners looking to deploy substantially large sums of capital in a single transaction.

### 7.6 Financial engineering

Investors can create value through three primary types of financial engineering: basis arbitrage, increasing leverage, and tax arbitrage.

Basis arbitrage: Through basis arbitrage, investors help a business lower its cost of debt capital to increase equity returns. Basis arbitrage can happen in multiple ways. Foreign investors, who acquire Indian RE assets, can refinance asset debt, and grow the portfolio with lower-cost debt capital obtained overseas. Domestic and foreign lenders are more comfortable lending to companies backed by reputable investors, particularly long-term institutional investors. For example, prominent Indian developers backed by foreign pension funds and sovereign wealth funds, Azure Power and ReNew Power, have raised foreign bonds at a significantly lower cost than domestic debt. Developers with a lower-cost capital than peers are in a stronger position to refinance old expensive debt, aggressively bid in new RE auctions to gain market share, acquire assets from distressed developers, and create new business offerings. Investors improve their risk profile by assembling a high-quality, diverse portfolio of operational assets with stable cash flows, attracting a strong management team, and establishing strong governance. This systematic reduction in risk then allows them to refinance asset debt at lower cost.

Increasing leverage: Increased asset leverage enables investors to enhance their equity returns. Leverage can be increased in two ways. First, increasing cash flows, by improving plant O&M or financially restructuring the holding vehicle, increases the total debt that an asset can service for a given leverage limit. Second, leverage limits can be increased for InvIT investments, by switching from a listed structure with a conditional 70 % leverage limit to an unlisted structure with no leverage limit.

Tax arbitrage: Investors can utilize tax arbitrage to increase equity returns by minimizing their tax burden. Placing assets in a tax-efficient vehicle like an InvIT minimizes tax burdens for developers and investors, particularly non-resident investors. Investors can also increase their return on equity by co-investing in RE projects with individuals or corporations seeking to benefit from accelerated depreciation (AD) incentives for development wind and PV assets. Indian tax law allows owners of PV modules, wind turbines, and specially designed devices running on wind energy, accelerated depreciation at a rate of 40 % [84]. This allows them to write off the asset value at an aggressive pace reducing their tax liability. This incentive encourages companies with taxable profits from other industries to invest in RE projects. Investors who place their RE asset SPVs in a Hold-Co can benefit from AD as follows. The hold-co can be owned by PE investors and an external investor with significant taxable income. The Hold-Co can hold RE asset SPVs and the external investor's sources of taxable income [84]. The AD of the RE assets can be offset by the external source of taxable income. AD becomes very relevant for balance-sheet financed projects on the Hold-Co's balance sheet and not on the underlying SPV's [89,124]. This is because Indian tax law does not allow AD in a SPV to be passed onto a SPV equity investor. In a common arrangement, a developer incorporates a Hold-Co, owned by the developer and an external investor, who has taxable income from other sources.

## 8 Outlook for new renewable energy value creation opportunities

Utility-scale RE project EIRRs are expected to decline over time with sector maturity and increased competition. Given this backdrop the following investment opportunities present emerging possibilities for investors and businesses to create new RE sector value across the power sector value chain. The authors expect increased entrepreneurial activity and PE capital flow into these less mature, emerging opportunity areas.

### 8.1 Asset consolidation

The recent trend of increasing asset consolidation will likely accelerate due to strong seller push and buyer pull drivers [125,126]. Over the last three years, sellers have been looking to unlock asset equity for various reasons. Some platforms have not been able to deliver an expected exit to their investors through a listing, InvIT, or selling the entire business. Now they need to return some capital or meet debt obligations. In some cases, investors may want to exit stagnant platforms that haven't delivered cash flow growth. Financially stressed platforms that have over levered themselves or have unmanageable DisCom payment issues, may need immediate liquidity. In other cases, platforms may be doing well, but want to sell older high tariff assets to avoid stranded asset risk, opportunistically sell during current high market valuations, and/or reinvest their equity capital in new growth opportunities. Concurrently, there has been strong buyer demand from new entrants looking to create critically sized asset portfolios and the largest developers, seeking to consolidate their market share and capitalize on their economies of scale.

Moving forward, NTPC and SECI will continue tendering larger and larger size auctions, with tariff ceilings that assume a low cost of capital that only large institutional backed players have access to. This will force smaller developers to sell assets that buyers will be looking to snap up.

### 8.2 Commercial and industrial segment

Presently Commercial and Industrial (C&I) segment PV plus wind capacity contributes 20 % of India's total combined PV plus wind capacity (of 6/2020) [75]. The recent uptick in investor activity in the C&I segment [98,106] is geared to accelerate as the segment has reached a growth inflection point—with corporate PPAs now cost competitive without government waivers on Open Access charges [127]. Moving forward, C&I RE capacity is expected to increase 4 × by 2025 due to favorable demand and supply economics driving the adoption of corporate PPAs [75]. On the demand side, C&I customers are strongly incentivized to reduce their grid consumption in favor of privately acquiring RE power: reduced lower electricity costs by 15–40 %, long–term cost predictability, comply with mandated Renewable Portfolio Standard requirements [82,128], and attain corporate sustainability goals. On the supply side, C&I projects offer investors and developers significant advantages versus utility-scale projects: higher equity returns, lower risk through diversification, a direct relationship with customers and large in-built growth prospects linked to their client's future RE adoption needs. C&I projects are the RE asset sub-class with the highest project returns: 12–15 % project IRRs and 15–20 % EIRRs [75]. C&I portfolios to date have enhanced credit profiles due to their distribution of risk across multiple, small scale assets and contracts with A to BBB rated corporations that have credit ratings higher than most DisComs. Through C&I projects, developers can directly contract with end-consumers versus selling powers to DisComs and waiting for DisCom payments. There is ample scope to directly negotiate C&I PPA tariffs, unlike hyper-competitive utility-scale reverse-auctions.

### 8.3 Oil and gas corporation synergistic partnerships

Four major global Oil and Gas Corporations, BP, Shell, Petronas, Total, have made sector RE investments in developer platforms [98]. Moving forward, an increasing number of Oil and Gas Corporations will likely enter the sector in response to growing activist and ESG shareholder pressure to diversify into RE [[129], [130], [131]]. Large Oil and Gas Corporations possess multiple strengths that will enable them to add synergistic value across the new frontiers of growth for RE companies: storage, the C&I segment, EV charging [75]. First, their global presence across the energy ecosystem, large capital base, and large in-house R&D gives them access to the latest storage and other incipient RE technologies like hydrogen. Second, their large, diverse supplier and partner network, gives them a customer acquisition advantage in the C&I segment, compared to domestic IPPs [75]. Third, their supply chain linked to the automobile industry, and global automobile service station networks, positions them to tap opportunities at the forefront of EVs and EV charging [75]. Fourth, their large capital base enables them to make large and aggressive opportunistic CAPEX investments across the Indian RE ecosystem.

### 8.4 Grid infrastructure

The pressing need to upgrade India's grid will likely create new PE grid investment opportunities in the future. Substantial cheap RE capacity is being added to India's grid that is not expanding at a corresponding pace [85] to accommodate it. Over the last decade, the compound annual growth rate of generation capacity, 9 %, has eclipsed that of transmission line growth, 5 %. Moreover, there has been a significant slowdown in annual growth of net transmission capacity: from 9 % in 2015 to 2.6 % in 2019 [16]. This slowdown is a consequence of underfunding and project delays [132]. Multiple Centre initiatives [133] to increase inter-state and intra-state transmission and distribution capacity, including the Green Energy Corridor to integrate 100 GW of RE, have turned out to be very disappointing [16,132,134]. Lower CAPEX by DisComs and transmission project development issues [3] have resulted in substantial reduction of grid investment as a fraction of total power sector investment from 50 % in 2010 to 30 % in 2020.

There has been significantly less PE activity in transmission than in RE [98]. This stems from the former's much more complex and longer duration development risks, the small number of active private developers in major transmission bids, and high barriers to entry for new developers. Moving forward, PE investors could play a role in buying operational assets from developers, to free up developer equity capital for new projects. Operational transmission assets, which have lower counterparty risk than RE assets [11,75,116], could be attractive for institutional investors seeking more stable investments.

### 8.5 Distribution operations

Should the Centre's efforts to privatize public DisComs prove successful [11], it will create distribution segment investment opportunities. The financially distressed public state DisComs are responsible for managing almost 95 % of India's distribution network. Eight private DisComs also operate, mainly serving urban areas, and demonstrate sustained profitability, superior power supply/quality, superior customer service, and low sub–15 % Aggregate Technical & Commercial losses [11,135]. Private DisComs have a proven track record of superior operational management and they primarily serve urban areas with miniscule exposure to agricultural consumers [11,19]. In the 2020 Covid-stimulus bill, the Centre announced its intention to privatize DisComs in all union territories (UTs) [11]. The measure has been introduced to template successful DisCom privatization in the states. The UT privatization initiative, however, has progressed at a slower-than-expected pace owing to country-wide protests from DisCom employee unions and numerous legal challenges [[135], [136], [137]]. Despite these challenges, the Ministry of Power has decided to continue privatization efforts and private sector's interest remains strong.

Buying distribution operations fits well with leading developers’ strategy of capturing increasing value across the power sector value chain [138]. DisComs are the exclusive interface with end-consumers and serve as the cash register for the entire power sector. If RE developers become the entity that controls end-consumers tariff setting and payment collection, they would be exceptionally positioned to capture the largest share of the power sector value chain [138]. Furthermore, today developers are dependent on financially distressed DisComs for PPAs. If developers themselves move into distribution, they can gain greater control over their PPA demand, eliminating offtaker risk, and capturing additional revenue through direct relationships with end-consumers.

It must be noted that privatizing DisComs will be a major challenge since the Centre lacks the constitutional authority to unilaterally impose mandates on the DisComs, and the significant adverse effects these strong reforms will have on major voting blocks.

### 8.6 Merchant power

Presently 88 % of India's generation volume [139] is covered through long term PPAs. Moving forward there is growing interest in alternative emerging procurement models [127] including spot-market transactions, shorter-term PPAs [140], the green term ahead market, real time trading, round the clock PPAs, short-term market derivatives, virtual PPAs, and interstate PPAs. Adoption of these new procurement models will result in new investment opportunities to generate value by repurposing RE and thermal power plants, particularly stranded assets, for merchant power [141] use.

## 9 Conclusion

The Centre has made bold climate commitments for 2030, including a 500 GW RE capacity target for 2030. $400 bn in new financing will be required to meet the 500 GW target. This amount dwarfs present RE sector capital flow. Given the scale of the financing challenge and the sector's complex risks, rapidly scaling-up growth and risk-friendly PE financing be critical to achieve India's target.

This research answers questions regarding the motivation, perceptions, strategies, and investment behavior of PE investors in the Indian PE sector. Presented answers to these questions have been distilled from primary research interviews with 40 executive-level sector practitioners and literature analysis. Systematic analysis of the key drivers behind PE investments and the strategic value creation themes for investors have been shared. Multiple global macroeconomic trends are driving PE investors and businesses to capitalize on Asian infrastructure opportunities. Within this opportunity set, Indian RE investments offer substantial value including EIRRs of 7–10 % USD, substantial market size, significant growth opportunity, fairly high liquidity and ideal ESG fit.

This research concludes that despite significant sector risks, investors remain confident they can achieve outsized risk-adjusted returns relative to most other global infrastructure assets. This optimism stems from investors' confidence in 1) their own ability to manage risk-return dynamics through judicious investment selection and investment management strategies, 2) the sector's uniquely large market size and demand, and 3) the Indian central government's perceived strong commitment to find creative solutions to chronic sector issues. Regarding 3), investors have tremendous confidence in the bankability of central offtakers SECI and NTPC and are motivated to pay a reasonable premium for assets contracted with them in order to mitigate their investment risks.

Despite the most meticulous investor calculus, outcome uncertainty and related investment risk cannot be completely eliminated. The findings presented here suggest investors’ best risk mitigation strategies include: buying high quality RE generation assets with bankable contracted offtakers, project portfolio diversification, an attitude of continuous learning, and partnerships with experienced management teams. In the final analysis, however, tail risks that could produce very large destructive investment outcomes, always remain. Such tail risks include the effects of Wars, grid Cyber attacks, Solar flares, future Covid-like Pandemics, and unexpected crippling supply chain shocks. The authors have found that awareness and study of such sector tail risks is lacking in the literature and industry. The sector would benefit significantly from future academic, government, insurance and business analysis of such tail risks.

Utility-scale RE project EIRRs are expected to decline over time with sector maturity and increased competition. Moving forward, this research finds that PE investors and businesses will have the opportunity to create new sector value by facilitating asset consolidation and partnering with companies that capture increasing value across the entire power sector value chain. These emerging possibilities include the C&I segment, grid infrastructure, synergistic partnerships with global oil and gas companies, distribution, and merchant power. The authors expect increased entrepreneurial activity and PE capital flow into these emerging opportunity-areas. The Central government would be well advised to study these areas—including their current market failures and regulatory/market barriers to new entrant activity and large capital formation—and initiate supportive policies of incoming private investment.

Findings drawn from the investment insights, themes and factor analyses discussed herein can be drawn upon when evaluating RE investments and policymaking worldwide. This research's primary finding that the Indian central government's strategy of using central agencies as intermediary electricity offtakers (*i.e*., credit sleeves) to mitigate state DisCom offtaker risk and increase investor confidence has been very successful. This has positive implications for similar emerging markets. Policy makers in emerging markets with political-economic and power sector features similar to those of India could use similar federal credit sleeve mechanisms to mobilize increased global private sector capital flows to their RE sectors. As discussed in Section 1.3, potentially applicable emerging markets include Mexico, Nigeria, Pakistan, Iraq, Nepal, and Malaysia.

To date, India's RE growth has been financed by various investors. PE investor activity has matured as the market has grown and evolving risks have become better understood. To meet India's 2030 climate commitments, the market will need to tap into new and underutilized global PE investors. There is certainly an opportunity for capital injection from new PE investors; since eight of the world's ten largest sovereign wealth and pension funds have yet to make sector PE investments. By providing stakeholders with a comprehensive understanding of current private investor activity, the authors hope to 1) make the RE sector more readily understandable and accessible to potential new investors and 2) inform better policy making decisions to attract new investments.

## CRediT authorship contribution statement

**Hemi H. Gandhi:** Writing – review & editing, Writing – original draft, Visualization, Methodology, Investigation. **Bram Hoex:** Writing – review & editing, Visualization, Supervision. **Brett Jason Hallam:** Writing – review & editing, Visualization, Supervision.

## Data availability statement

Data included in article/supplementary material is referenced in the article.

## Funding

Hemi H. Gandhi acknowledges personal fellowship support from the Harvard University Frank A. Knox Traveling Memorial Fellowship, Harvard University John A. Paulson School of Engineering and Applied Sciences Associate position, and the Australian American Association Graduate Education Scholarship Award.

## Declaration of competing interest

Authors declare that they have no known competing financial interests or personal relationships that could have appeared to influence the work reported in this paper.

## Footnotes

1 See Supplemental Materials for a complete list of all abbreviations used herein.

2 See Supplemental Materials Table I for an explanation of the policies and market forces behind India's rapid RE growth since year 2000.

3 India has a federalist system of government which exists as a union amongst the Central government (the Centre), 29 states, and 8 Union Territories.

4 Typically investors factor in a 4–5% yearly INR depreciation against the US dollar in their modeling. This assumption has been reasonably consistent with the Rupee depreciation [76] over the last decade.

5 Stranded asset risk is still significant for older RE assets with high-tariff PPAs. Potential buyers often avoid these assets due to future offtaker and curtailment risks.
