# Supplementary material for: Private equity renewable energy investments in India
Source: Heliyon. 2024 Dec 14;11(1):e41098. doi: 10.1016/j.heliyon.2024.e41098 (PMC11732484; doi:10.1016/j.heliyon.2024.e41098)
Supplement: Multimedia component 1 [file mmc1.docx]

**Abbreviations:**

Basis Point (bp)

Billion (bn)

Central Government (Centre)

Commercial and Industrial (C&I)

Distribution Companies (DisComs)

Debt-Service-Cover-Ratio (DSCR)

Earnings Before Interest Taxes, Depreciation, and Amortization (EBITDA)

Engineering Procurement Contracting (EPC)

Environmental Social and Governance (ESG)

Equity Internal Rate of Return (EIRR)

Foreign Direct Investment (FDI)

Gigawatt (GW)

Great Financial Crisis (GFC)

High-Net-Worth-Individual (HNWI)

Holding Company (Hold-Co)

Kilo-Watt-Hour (kWh)

Independent Power Producer (IPP)

Indian Rupee (INR)

Infrastructure Investment Trust (InvIT)

Initial Public Offerings (IPO)

Merger and Acquisition (M&A)

Mergers and Acquisitions (M&A)

Million (mn)

Operations and Maintenance (O&M)

Operating Profit Before Depreciation Interest Tax and Amortization (OPBITA)

Photovoltaic (PV)

Power Purchase Agreement (PPA)

Power Sale Agreement (PSA)

Private Equity (PE)

Real Estate and Investment Trust (REIT)

Renewable Energy (RE)

Securities and Exchange Bureau of India (SEBI)

Solar Energy Corporation of India (SECI)

Special Purpose Vehicle (SPV)

United Sates Dollar (USD)

Union Territory (UT)

**SUPPLEMENTARY MATERIAL**

**Table I.** Policies and market forces behind India’s rapid RE capacity growth and tariff reductions. Information from main manuscript references [5,21,84].

| **Category** | **Driver** | **Description** | **Impact / Challenges** |
| --- | --- | --- | --- |
| **Demand Generative Policies** |  |  |  |
|  | **175 GW RE Target**  *(Year 2022)* | - In 2015, the Centre announced an ambitious target of 175 GW of RE by 2022. - This equated to increasing RE capacity four fold in just seven years. - The target comprises 40 GW rooftop PV, 60 GW utility-scale PV, 60 GW wind, 10 GW biomass, 5 GW small hydro. | - The target signals the Centre’s long-term commitment to the RE sector. - The target signals a large, long-term project pipeline to domestic RE ecosystem and foreign investors. |
|  | **500 GW RE Target**  *(Year 2030)* | - In 2021, the Centre announced a world leading 500 GW RE target by 2030. - The target includes 300 GW PV and 140GW wind sub-targets. | - The target signals the Centre’s long-term commitment to the RE sector. - The target signals a large, long-term project pipeline to domestic RE ecosystem and foreign investors. |
|  | **Large Federal Auction Tenders** | - Large federal auction tenders have created a huge demand pipeline for RE projects. - Moving forward, Central offtakers SECI and NTPC are expected to bid at least 8–10 GW of projects per year. | - This large auction pipeline incentivizes investors and developers to invest in the sector for the long term. - India has become the world’s largest RE auction market (cumulatively until 2019). - Central offtaker insistence on low-tariff ceilings and developer concerns of inadequate transmission capacity have resulted in several auctions being partially or fully cancelled, post-bidding. These challenges create uncertainty for participating developers. |
|  | **Renewable Purchase Obligations (RPOs)** | - Since 2011, the Centre has mandated states to purchase a specified minimum portion of total contracted electricity from RE source in the form of Renewable Purchase Obligations (RPOs). - These RPOs apply to DisComs and large C&I consumers. - Renewable Energy Certificates (REC) can be purchased to satisfy RPOs. - RPO requirements have gradually risen each year and will reach 21% by 2022. | - In the early 2010s, when RE sources were significantly less competitive than thermal power, RPOs created demand certainty at the state-level. - RPO enforcement is incredibly weak. - RPO compliance varies widely between states, from 177% in Karnataka to 2% in West Bengal. - Most RE capacity is very concentrated in western and southern India. States with low RE capacity are constrained to purchase RECs, on top of existing thermal power commitments. In such states, financially distressed DisComs do not comply with RPOs. |
| **Financial Incentives Policies** |  |  |  |
|  | **100% FDI Without Review** | - RE Sector FDI up to 100% ownership is allowed without governmental review or approval. | - This concession makes it easier for foreign pension funds, sovereign wealth funds, infrastructure funds, and oil and gas majors to invest in the sector. |
|  | **Accelerated Depreciation (AD)** | - PV and Wind projects are allowed AD of 40% in the first year of commissioning. | - AD reduces project investors’ tax burden. - The AD can be claimed against income from outside the sector. This incentivizes a diverse array of corporations and HNWIs with taxable profits to diversify into RE investments. - AD incentives were key drivers for wind deployment prior to 2015. |
|  | **Lower Corporate Tax Rate** | - The tax-rate for RE generators is 17.2%, compared to 34.6% for other corporates. | - This reduces PV and wind LCOEs by an estimated 8% and 10%, respectively. |
|  | **ISTS Waiver** | - PV/Wind projects commissioned between 2016-2022 are exempted from Inter-State Transmission System (ISTS) Charges | - This concession reduces the delivered cost of electricity to an offtaker by up to 0.65 Rs/kWh, making RE projects more cost-competitive with thermal power. |
| **Investment Risk Mitigating Polices** |  |  |  |
|  | **Solar Parks** | - In dedicated central and state solar parks, government agencies are responsible for acquiring large contiguous land zones land and building grid connection infrastructure. | - This reduces project development / curtailment risks. |
|  | **Federal auctions** | - In federal auctions, federal offtakers SECI and NTPC act as intermediate procurers on behalf of financially distressed state DisComs. - This protects developers from DisCom payment delays and gives them a quasi-sovereign payment guarantee. | - These auctions reduce investor offtaker risk. - Federally auctioned awarded capacity has overtaken that of state auctions since 2018. |
|  | **Green Energy Transmission Corridor** | - In 2013 the Centre launched the Green Energy Transmission Corridor (GEC) program to create a dedicated transmission network for large-scale RE generation. - The GEC’s goal is to enhance inter-state and intra-transmission to evacuate power from RE-rich states (with surplus capacity) to energy-deficit states. - The first GEC project (a $5 bn 1,800 km HV transmission line) began in 2018. | - The Corridor, if successful, will reduce Project Development and Curtailment Risks. . - General and GEC-specific transmission capacity expansion, however, has been significantly slow. - India’s annual net transmission capacity growth rate has decreased from 9% (FY 15­–16) to 2.6% (FY 19–20) - Due to DisCom underinvestment, the ratio of grid investment/total power sector investment has declined from 50% to 30% between 2015-2020 - Major recently built RE plants have been left with inadequate evacuation infrastructure, crippling developer cash flows. - Lack of new transmission capacity has been a major factor for low participation in several mega PV and wind auctions. |
|  | **“Must-Run Status” for RE plants** | - The Indian Electricity Grid Code 2010 grants RE sources priority dispatch ahead of other generation sources. - Indian grid operators are required to offtake all RE generation regardless of cost, generation amount, or time-of-day. | - This mandate educes Curtailment Risk. - RE curtailment is only allowed in event of grid technical constraints. States increasingly exploit this provision as a loophole to curtail expensive RE sources on “technical” grounds. |
| **Market Forces** |  |  |  |
|  | **Rapid Reduction in Hardware Costs** | - Between 2011–2020, global spot PV module and wind turbine prices, fell   $0.93/W🡪$0.18/W and $1,500/kW🡪 $885/kW, respectively.   - These price reductions have been driven by economies of scale, technology improvements, and intense manufacturing competition. | - These declining project capital expenditure costs have contributed to declining tariffs. |
|  | **Decreasing project finance costs** | - Indian RE project debt costs have reduced (domestic debt 13% 🡪 9% from 2011–2020) due to accommodative monetary policy and a lower sector risk premium. | - These declining project finance costs have contributed to declining tariffs and portfolio value accretion for developers. |
|  | **Extreme developer competition** | - Extreme competition exists amongst domestic and foreign developers. - Major players are focused on establishing a large portfolio base to get market-share and scale. - Concurrently, several new foreign entrants and control platforms regularly enter the market every quarter. - Competitive tariff bidding has accelerated since the transition from the Feed-In-Tariff regime to reverse-bidding auction in 2016s. In these real-time auctions, developers have to bid very aggressively to win. | - This extreme competition has led to declining tariffs. - Competition has resulted in developer project cost optimization. - An environment of rapidly declining tariffs has increased Stranded Asset Risk and Offtaker Risk. DisComs have an incentive to delay signing PPAs, cancel letters of award, and renegotiate PPAs. Furthermore, they are undeterred from routinely breaching contracts because of poor legal contract enforceability. |

**Table II. Characterization of critical Indian RE sector investment risks.** Information from main manuscript reference [21].

| **Risk Type** | **Description** |
| --- | --- |
| **Project Development Risk** | - This comprises the set of risks associated with developing a greenfield project from winning PPA contracts to delivering an operational asset. - Such risks include cost-overruns, time over-runs, and project completion failure. - Components of these risks include issues related to project financing, land acquisition, plant construction, arranging adequate transmission evacuation infrastructure and obtaining necessary permits. |
| **Offtaker Risk** | - The most significant risk to Indian RE investments is Offtaker Risk, where an offtaker breaches its contractual obligations - There are three types of Offtaker Risk:   - PPA signing delay/cancelation   - Payment delay   - PPA renegotiation/cancelation - DisComs are incentivized to pursue these tactics due to reduce their financial distress in an environment of rapidly falling RE tariffs. Furthermore, they are undeterred from routinely breaching contracts because of poor legal contract enforceability. |
| **Stranded Asset Risk** | - Stranded RE assets are operational assets that suffer premature devaluations or write-downs due to cash flow losses and lack of buyers. - Potential buyers may avoid such an asset out of fear of Offtaker Risk or Curtailment. - Such situations leave investors stranded with no ability to recycle invested capital into more lucrative investments. |
| **Volume Risk** | - Plant generation volume loss, relative to its projected generation, can occur due to inaccurate initial assumptions about weather and hardware generation efficiency over time. - Hardware can perform sub–optimally due to degradation and sub–optimal cleaning from poor practices or water scarcity. - Volume loss represents irrecoverable loss of project cash flows for the generator. |
| **Curtailment**  **(Volume/Offtaker Risks)** | - Curtailment is an involuntary reduction in a generator’s output due to the grid operator restricting electricity delivery from the generator to the grid. - Curtailment irrecoverably impairs project cash flow and undermines the generator’s timely ability to service debt. - Technical curtailment can occur due to transmission congestion, lack of transmission access, excess generation during low-demand periods, and frequency requirements. - Commercial curtailment can be financially motivated to reduce DisCom costs (implemented via DisCom collusion with state grid operators). There is increasing evidence of commercial curtailment being selectively enforced on older high tariff RE sources, done under the guise of maintaining grid stability to circumvent the RE must–run status. |
| **Regulatory Risk** | - Unexpected regulatory changes-in-law can detrimentally impact expected investor returns. Recent major examples of such changes–in–law include: The Goods–and–Service–Tax (GST), 40% customs duty on imported PV modules, and new Open Access charges. |
| **Inflation Risk** | - Unexpectedly high inflation is a significant and systematically underestimated sector risk. - High inflation can damage realized EIRRs through increasing plant CapEx and debt servicing costs. - Sector PPAs are vulnerable to inflation risk because they are fixed-price PPAs with (0–4%) annual escalation not linked to inflation. |
| **Exchange Rate Risk** | - Investors are exposed to Exchange Rate Risk if there is a mismatch between their currency of ultimate interest (for debt servicing or pension payments) and the Rupee, in which they harvest RE asset cash flows. - In their financial models, most investors assume 4-5% annual INR depreciation against the dollar, comparable to observed depreciation over the last decade. Higher rupee depreciation over a plant’s life due to high Indian economic growth and/or high inflation, will compromise investor returns. |
| **Tail Risk** | - RE assets face investment tail risk due to cyberattacks and electromagnetic pulses triggered by a solar geomagnetic storm or a manmade thermonuclear denotation. - Such events can severely impair or shut down the grid, producing widespread offtaker default under PPA *force majeure* clauses. |
